# Cases in Obstetric Practice

**Published:** 1846-04

**Authors:** W. Franklin Barr

**Affiliations:** Greenville, Tenn.


*Cases in Obstetric Practice.* By W. Franklin Barr, M. D., of Greenville, Tenn.—I. Dysmenorrhœa.—*A case cured with Extract Stramonium and Prussiate of Iron.*—I was called to see Mrs. G., a middle aged woman, the mother of seven children, spare make, and of laborious habit. I found her laboring under the following symptoms: Violent pain in the head; dry skin; pulse of natural size, but frequent; bowels constipated; tongue covered with white fur, with clean edges; pains in the back, hips, loins, thighs, and hypogastrium, resembling those of parturition—which were followed by profuse discharge, that would debilitate her very much, but which relieved her of pain until another paroxysm. Each discharge would be preceded by the pains mentioned above of a character, and which, in her own language, were "as severe as those attending the birth of any child she ever had."

She had been laboring under this affection for eight or nine months; the catamenia returning at irregular periods of three or four weeks. During the first two or three menstrual periods, the discharge was accompanied with coagula, at the fourth with a pseudo-membranous substance, (according to her account,) "about the size of a placenta." Since then, no coagula or membranous substance has accompanied the discharges. Her general health was much impaired,—so much so that she had been unable to attend to her work for sometime. The lumbar vertebræ were tender on pressure; pubic region tender during menstrual period.

She had been under the attention of another physician—I being requested by him to visit her when I did, as he was unable to go himself. I found, from the medicine left by him that he was treating her for menorrhagia. He had given opium and acetate of lead, to be taken at each menstrual stage; and nothing in the *interval.* As this remedy relieved the pain and checked the discharges, I requested her to continue it—only leaving some purgative medicine, as her bowels were constipated, and recommended a blister to the lumbar vertebræ.

As the attending physician intended leaving the country, the case was placed in my care. To effect a cure, I prescribed Dewees' laxative pills to keep the bowels in a soluble condition, five grains of prussiate of iron three times a day, and four days previous to the return of the attack, one-fourth of a grain of the extract of strmonium, three times a day, until it produced vertigo, when it was to be discontinued until this subsided, when it was to be resumed. If the pains were severe on the return of the catamenia, 8 grains camphor and 4 grains pulv. Doveri, every two hours until relief was given. Flannel was directed to be worn next the skin. As there were symptoms of prolapsus uteri, directed recumbent position, injection decoction red oak bark, and the T bandage

The *first* return of the catamenia after using the above remedies was attended with no more pain than she had experienced before marriage, and no more than generally falls to the lot of females at this time. The symptoms of procedentia were also removed.

The remedies were continued for about four months, when

I ceased my attention. Her general health is now good, and she has become somewhat fleshy.

II. *Ergot in Uterine Hemorrhage.*—May 1st. I was called to see Mrs. C.—she had been laboring under uterine hemorrhage for a day. Her husband, being a physician, had prescribed for her himself. He had given ipecacuhana by itself, made applications to the vulva, &c. Her bowels had been operated upon by a cathartic. I prescribed opium, acetate of lead, and ipecac. in combination. This was continued for two days, but seemed to do but little good. I then prescribed ergot in doses of 10 grains every four hours. There was no return of the hemorrhage after the first dose had been given. She recovered without taking another dose. —*S. Med. & Surg. Jour.*

